# The value of urinary gonadotropins in the diagnosis of central precocious puberty: a meta-analysis

**DOI:** 10.1186/s12887-022-03481-1

**Published:** 2022-07-28

**Authors:** Dan Xu, Xueying Zhou, Junfei Wang, Xi Cao, Tao Liu

**Affiliations:** 1 Department of Pediatrics, the First Affiliated Hospital of Nanchang University, Jiangxi 330006 Nanchang, China; 2 Department of Pediatrics, The Fifth Affiliated Hospital, Sun Yat- sen University, New Xiangzhou, Zhuhai, Guangdong Province 519000 China

**Keywords:** Urinary gonadotropins, Luteinizing hormone, Follicle-stimulating hormone, Gonadotropin-releasing hormone stimulation test, Central precocious puberty

## Abstract

**Background:**

The gonadotropin-releasing hormone (GnRH) stimulation test is time-consuming, invasive, and costly. However, it is the diagnostic gold standard for central precocious puberty (CPP), which in girls is defined as the onset of secondary sexual characteristics before the age of 8 years accompanied by breast buds, accelerated growth, and advanced bone age. This meta-analysis was performed to compare the diagnostic value of urinary gonadotropins and the GnRH stimulation test for CPP.

**Methods:**

We searched six databases for relevant literature. In accordance with the Preferred Reporting Items for Systematic Reviews and Meta-Analyses guidelines, we estimated the sensitivity, specificity, area under the summary receiver operating characteristic curve (AUC), and publication bias.

**Results:**

Six eligible trials fulfilled the inclusion criteria. In the meta-analysis of urinary luteinizing hormone (ULH), after excluding the data of one study, we obtained an AUC of 0.90 (sensitivity = 0.81, specificity = 0.85). The meta-analysis of the ULH to urinary follicle-stimulating hormone (UFSH) ratio revealed an AUC of 0.8116 (sensitivity = 0.79, specificity = 0.84).

**Conclusion:**

Both the ULH level and ULH:UFSH ratio are effective and available approaches for CPP diagnosis.

**Trial Registration:**

INPLASY 2021120076.

**Supplementary Information:**

The online version contains supplementary material available at 10.1186/s12887-022-03481-1.

## Background

Puberty is a complex progression of hormonal alterations leading to the achievement of mature reproductive capacity. The onset of puberty is activated by pulsatile release of gonadotropin-releasing hormone (GnRH). With the development of the social economy and improvements in living conditions, the age at pubertal onset has advanced worldwide, and the incidence and morbidity of precocious puberty is increasing annually [1–3]. Precocious puberty is more common in girls than in boys. In girls, the onset of secondary sexual characteristics before the age of 8 years is considered precocious puberty. It can be divided into two kinds, central precocious puberty (CPP) and peripheral precocious puberty [4]. CPP may result in accelerated growth and an early age at menarche, and then it would lead to a decreased final adult height and some psychological and health problems in adulthood, taking diabetes, cardiovascular disease for example [5, 6]. Therefore, in the long run, early and accurate diagnosis of CPP is especially significant. In addition to examinations of secondary sexual characteristics and bone age, the GnRH stimulation test has been indispensable in the diagnosis of CPP. However, this test, while requiring 3 collections (0, 30 and 60 min), is often done with 5 samples, including 90 and 120 min time points. Not only is the GnRH stimulation test invasive, time-consuming, and expensive, but patient cooperation is also sometimes difficult. To explore convenient and accurate diagnostic procedures for CPP, many researchers have assessed the value of urinary gonadotropins from urinary samples, including first-voided urine samples and random urine samples. Both types of samples have been used to evaluate the levels of urinary luteinizing hormone (ULH) and urinary follicle-stimulating hormone (UFSH). First-voided urine is collected from patients who were informed to empty their bladder before going to bed and to refrain from voiding until the next morning. Random urine samples are collected at any time during the period of the GnRH stimulation test. To date, many authors have found that urinary gonadotropin measurements are a potential alternative approach for the diagnosis of CPP. However, this remains a controversial issue because of the absence of unified standards and evidence-based support for this approach.

Nocturnal ULH and UFSH can represent gonadotropin excretion in children with normal and early puberty [7]. Therefore, this meta-analysis was performed to assess the value of first-voided ULH and the ratio of ULH to UFSH in the diagnosis of female CPP and to compare the accuracy between urinary gonadotropins and serum GnRH-stimulated gonadotropins.

## Methods

This meta-analysis is reported in accordance with the Preferred Reporting Items for Systematic Reviews and Meta-Analyses (PRISMA) guidelines. The protocol for this systematic review was registered on INPLASY (registration number: INPLASY 2021120076) and is available in full on inplasy.com (https://doi.org/10.37766/inplasy2021.12.0076).

### Literature search

We searched the databases of PubMed, Embase, Cochrane Library, Web of Science, China National Knowledge Infrastructure (CNKI), and Wanfang for relevant literature published until 7 December 2020 in all languages. The search was performed using a combination of keywords and free words. The keywords were “puberty, precocious,” “urinary luteinizing hormone,” and “urofollitropin.” According to the PRISMA diagnostic test accuracy guidelines, the keywords regarding research methods were “sensitivity” and “accuracy.” Each keyword and its free words were combined with “or.” The different keywords were combined with “and.”

### Study selection

The inclusion criteria were as follows.

1. The study was designed as a diagnostic test study, and the results had been published.
2. All patients in the study were female with Tanner stage ≥ 2 breast development, advanced bone age by ≥ 1 year, and accelerated growth.
3. Urine and serum samples were collected for gonadotropin measurement on the same day for each patient.
4. First-voided urine was used for all urinary samples. For reliable evaluation of urinary gonadotropins, all patients had been informed to empty their bladder before going to bed and to refrain from voiding until the next morning.
5. The gold standard was a serum LH level of ≥ 5 mIU/L or a serum LH:FSH ratio of > 0.6.

Studies that did not meet the inclusion criteria were excluded. Additional exclusion criteria were fundamental experimental studies, such as animal studies; reviews, conference reports, repeatedly published studies, summary articles, and case reports; and studies without sufficient data.

### Literature quality assessment

Literature quality assessments were performed by two independent investigators. They independently extracted and incorporated the data. When disagreements arose, the investigators discussed the study until a consensus was reached. The assessment was performed based on the Quality Assessment of Diagnostic Accuracy Studies 2 (QUADAS-2) [8].

### Data extraction

Microsoft Excel 2010 (Microsoft Corp., Redmond, WA, USA) was used for data extraction. The following relevant information required for this meta-analysis was extracted: first author, publication year, nationality, sex, age, ethnicity, numbers of participants in CPP group and control group, gold standard test results, and sensitivity, specificity, true positive, false positive, true negative, false negative, and accuracy measures [9].

### Statistical analysis

In this article, Meta-DiSc version 1.4 (http://www.hrc.es/investigacion/metadisc_en.htm) was used to evaluate threshold effects and heterogeneity. We performed Cochran’s Q test to assess heterogeneity and used the inconsistency index (I^2^ test) to assess the magnitude of heterogeneity among studies. If the *P*-value of Cochran’s Q test was > 0.1 and the I^2^ value was < 50%, no heterogeneity was present, and a fixed-effects model was performed. If the *P*-value was < 0.1 or I^2^ was > 50%, great heterogeneity was present, and a random-effects model was performed. The pooled sensitivity, pooled specificity, pooled diagnostic odds ratio, pooled positive likelihood ratio (PLR), and pooled negative likelihood ratio (NLR) were all calculated by Meta-DiSc, and the *P*-values and 95% confidence intervals (CIs) were calculated at the same time. The area under the summary receiver operating characteristic curve (AUC) was also calculated. Sensitivity evaluation and publication bias were performed with STATA version 15.0 (StataCorp, College Station, TX, USA) [10]. If > 10 articles were included in this meta-analysis, we applied Deeks’ funnel plot to assess the extent of potential publication bias. Otherwise, we used the Begg rank correlation test and Egger test to analyze publication bias.

## Results

### Baseline characteristics of included studies

**Fig. 1:**
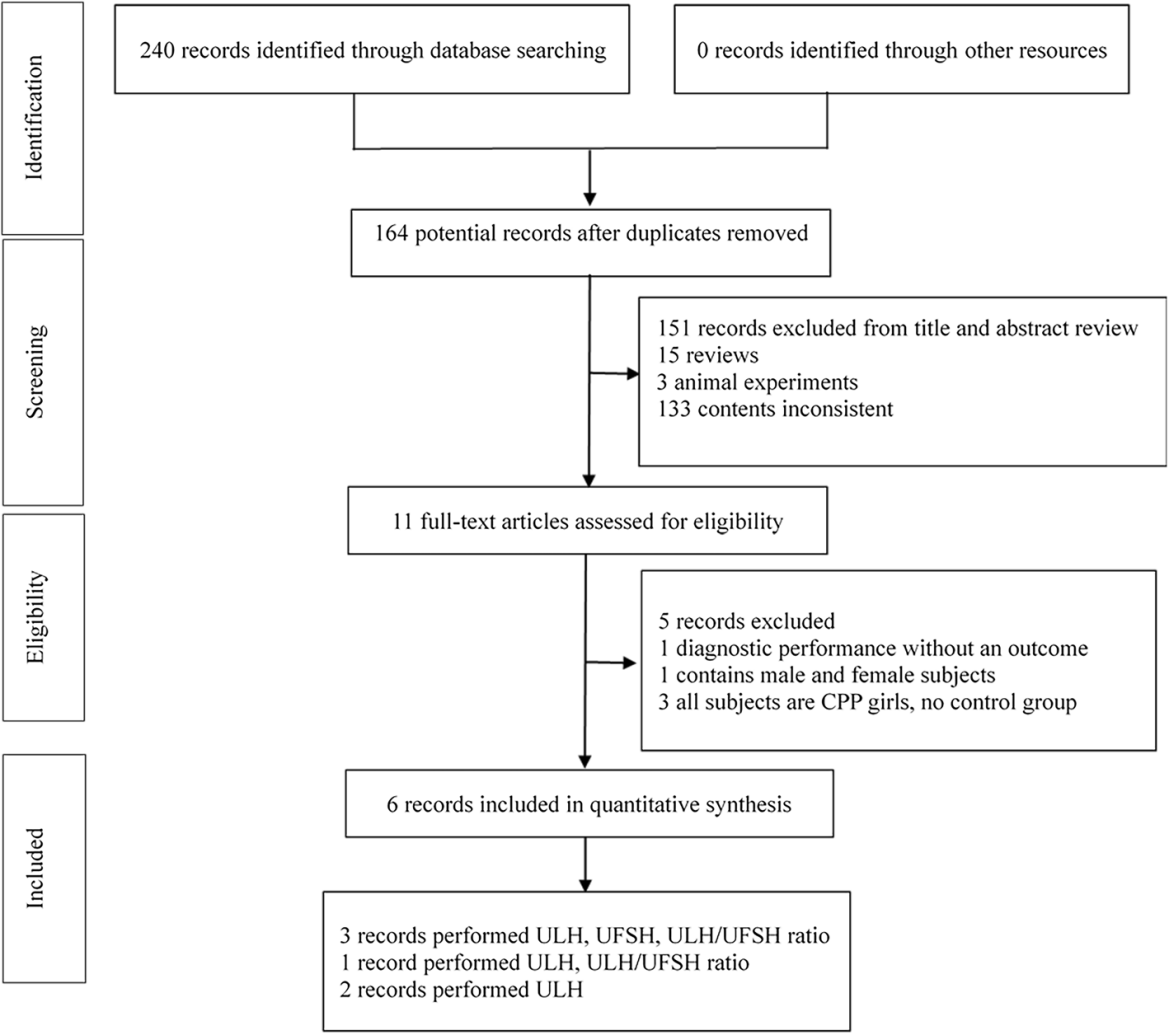
Study selection

We identified 310 candidate studies published before 7 December 2020. Considering that the prevalence of true precocious puberty has changed over the years, 70 studies published before 2000 were excluded. After reviewing the titles and abstracts of the remaining 240 studies, we deleted 76 duplicate studies; 15 reviews, editorials, or systematic analyses; 3 animal experiments; and 133 studies with irrelevant content. Thus, 11 full papers were reviewed. Finally, the eligibility criteria were fulfilled by 6 studies [11–16] involving 491 participants (Fig. 1). Potential bias was identified for all of the included studies. The main sources of bias were index tests, which might have introduced systematic error. The six trials were all published from 2012 to 2019 (three were published in 2019). The baseline characteristics of the included studies are shown in Table 1. Among the six included studies, diagnostic testing by ULH was assessed in five studies [11–14, 16]. Diagnostic testing by the ULH/UFSH ratio was performed in five studies, but one did not use first-voided urine; therefore, four studies [11, 13, 15, 16] were ultimately assessed. We did not assess the diagnostic value of the UFSH level for CPP because many studies have indicated that the serum FSH level alone does not have diagnostic significance.

**Table 1:** Baseline characteristics of eligible studies

Author (Year)	Country	Gold standard	Serum Gn	Urinary Gn	UGn corrected or not	Inclusion criteria	Sample size	Age (y)	Blind or not
Shim, YS 2019 [11]	Korea	GnRH ECLIA Stimulation test^a^		DELFIA	No	Tanner breast stage ≥ 2; an advanced bone age (BA) by > 1 year	100	6.0–8.9	Yes
Kolby, N 2017 [12]	Denmark	GnRH Stimulation test	IFMA	IFMA	Yes	Tanner breast stage ≥ 2	25	< 8.0	Yes
Yang, QH 2019 [13] (supplement 1)	China	GnRH Stimulation test	Not mentioned	CLIA	No	Tanner breast stage ≥ 2; an advanced BA by ≥ 1 year; Accelerated growth	184	6.0–10.0 (6.7 ± 0.5)	Yes
Chen, Y 2016 [14] (supplement 2)	China	GnRH Stimulation test	Not mentioned	ICA	Yes	Tanner breast stage ≥ 2; an advanced BA by ≥ 1 year; Accelerated growth	70	< 10.0 (7.12 ± 1.99)	Yes
Ma, XY 2019 [15] (supplement 3)	China	GnRH Stimulation test	CMA	ICA	Yes	Tanner breast stage ≥ 2; an advanced BA by ≥ 1 year; Accelerated growth	49	4.0–8.0 (7.41 ± 1.48)	Yes
Zhang, TT 2012 [16] (supplement 4)	China	GnRH Stimulation test	ICMA	ICMA	No	Tanner breast stage ≥ 2; an advanced BA by ≥ 1 year	63	(8.44 ± 1.20)	Yes

*Gn* gonadotropin, *UGn* urinary gonadotropin, *GnRH* gonadotropin-releasing hormone, *BA* bone age, *ECLIA* electrochemiluminescence immunoassay, *DELFIA* dissociation-enhanced lanthanide fluorescence immunoassay, *IFMA* immunofluorometric assay, *CLIA* chemiluminescence immunoassay, *ICA* immunochromatography assay, CMA chemiluminescence, *ICMA* immunochemiluminescence assay

^a^Diagnosis: serum luteinizing hormone peak of ≥ 5.0 mIU/mL or serum luteinizing hormone:follicle-stimulating hormone ratio of > 0.6

The first included study not only evaluated the first-voided urinary gonadotropin levels but also tested random urinary gonadotropin levels; however, we only collected the first-voided urine results [11]. Only the value of ULH was examined in the second included study. In the fourth study, first-voided urine samples were obtained on the same day as the GnRH stimulation test and the day before that to examine urinary LH level twice [14]. In this study, the ULH:UFSH ratio was calculated using 4-h urine samples during the GnRH stimulation test period. Therefore, in accordance with the inclusion criteria, this meta-analysis collected the sensitivity and specificity of ULH on the day that the stimulation test was conducted. The fifth study included only the ULH:UFSH ratio; this was a 6-month follow-up study of girls with premature thelarche, and GnRH stimulation tests were performed at the beginning and end of the study period [15]. Ultimately, we included the data after the 6-month follow-up, which were used to differentiate between CPP and premature thelarche. Half of the control groups in these studies included girls without precocious puberty, and half included girls with premature thelarche. The meta-analysis of the diagnostic value of ULH for CPP was performed using five records (nos. 1, 2, 3, 4, and 6), and the meta-analysis of the diagnostic value of the ULH:UFSH ratio for CPP was performed using four records (nos. 1, 3, 5, and 6).

### Meta-analysis of urinary LH for diagnosis of CPP

All relevant information from the five included studies is shown in Table 2.

**Table 2:** Relevant information for urinary luteinizing hormone from included studies

Author	Sensitivity	Specificity	TP	FP	FN	TN	Cut-off value
Shim, YS	91.90(%)	63.20(%)	57	14	5	24	0.58 IU/L
Kolby, N	75.00(%)	92.31(%)	9	1	3	12	> 2SD
Yang, QH	76.81(%)	90.43(%)	53	11	16	104	1.60U/mmol
Chen, Y	71.40(%)	76.10(%)	26	8	11	25	1.43U/mmol
Zhang, TT	71.40(%)	90.50(%)	30	2	12	19	0.113 IU

*TP* true positive, *FP* false positive, *FN* false negative, *TN* true negative

> 2SD: urinary LH concentration that is 2 standard deviation higher than the average concentration of same age and same sex

#### Threshold effect

In Meta-DiSc version 1.40, the Spearman correlation coefficient between the sensitivity logarithm and the (1 − specificity) logarithm was 0.30 (*P* = 0.62 > 0.05), indicating that there was no threshold effect. Furthermore, by drawing the symmetrical summary receiver operating characteristic curve, there was no “shoulder-arm shape,” which further illustrated that there was no threshold effect.

#### Heterogeneity and inconsistency assessments

**Fig. 2:**
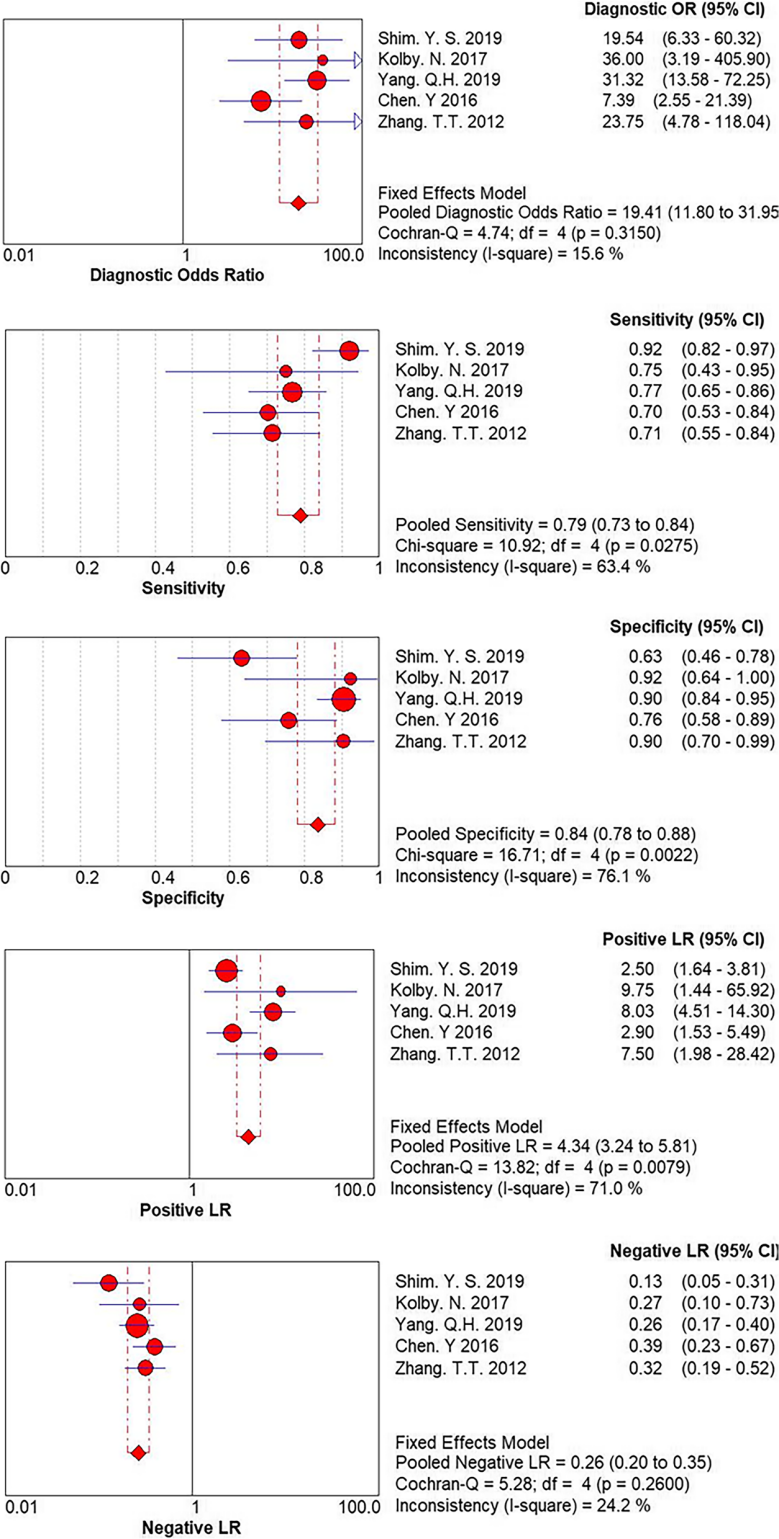
Q test and I^2^ statistic within a visual forest plot for ULH

**Fig. 3:**
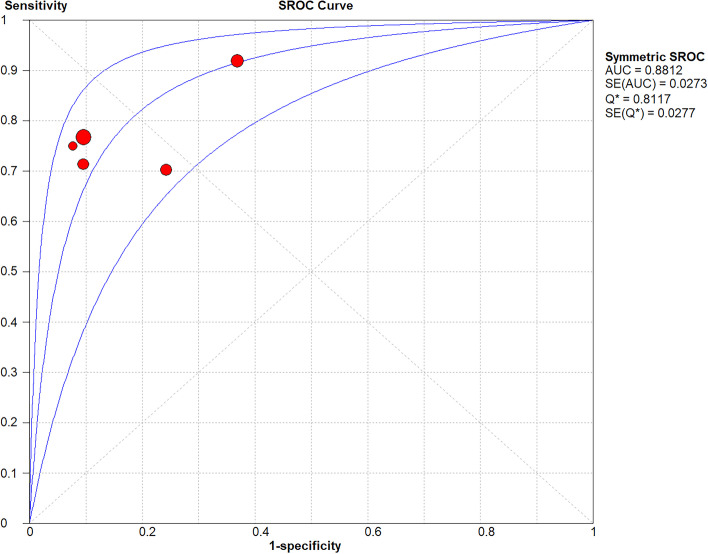
Evaluation index of diagnostic test for ULH

Based on a *P*-value of Cochran’s Q test of > 0.1 and I2 value of < 50%, a fixed-effects model was used (Fig. 2). Forest plots of the pooled sensitivity, pooled specificity, and pooled PLR and NLR are presented in Fig. 2: pooled sensitivity = 0.79 (95% CI = 0.73–0.84), pooled specificity = 0.84 (95% CI = 0.78–0.88), pooled PLR = 4.34 (95% CI = 3.24–5.81), and pooled NLR = 0.26 (95% CI = 0.20–0.35). The AUC was 0.8812, and the Q index was 0.8117 (Fig. 3).

#### Sensitivity analysis

The sensitivity analysis illustrated that three studies in this meta-analysis might have caused bias, which referred to Shim et al.’s [11], Yang et al.’s [13] and Chen et al.’s studies [14]. Subsequent sensitivity analyses were therefore conducted, and the results showed that the fourth record had the largest effect (Table 3).

**Table 3:** Sensitivity analysis of outcomes by excluding trials with a high risk of bias

Author	Sensitivity	Specificity	PLR	NLR	AUC
Shim, YS	0.74	0.88	5.91	0.29	0.7559
Yang, QH	0.80	0.76	3.30	0.27	0.8661
Chen, Y	0.80	0.85	4.75	0.24	0.9035

*PLR* positive likelihood ratio, *NLR* negative likelihood ratio, *AUC* area under the curve

#### Publication bias

Only five articles were included in the meta-analysis of ULH; therefore, we applied the Begg rank correlation test (*P* = 0.81) and Egger linear regression test (*P* = 0.96), which indicated that no publication bias existed.

### Meta-analysis of ULH:UFSH ratio for diagnosis of CPP

All relevant information from the four included studies is shown in Table 4.

**Table 4:** Relevant information for urinary luteinizing hormone: follicle-stimulating hormone ratio from included studies

Author	Sensitivity	Specificity	TP	FP	FN	TN	Cut-off value
Shim, YS	67.70(%)	81.60(%)	42	7	20	31	0.13
Yang, QH	89.85(%)	96.52(%)	62	4	7	111	0.845
Ma, XY	80.00(%)	55.60(%)	20	11	5	13	0.512
Zhang, TT	76.20(%)	52.40(%)	32	10	10	11	0.044

*TP* true positive, *FP* false positive, *FN* false negative, *TN* true negative

#### Threshold effect

Meta-DiSc version 1.40 showed that the Spearman correlation coefficient between the sensitivity logarithm and the (1 − specificity) logarithm was − 0.40 (*P* = 0.60 > 0.05), indicating that no threshold effect existed. The symmetrical summary receiver operating characteristic curve showed no “shoulder-arm shape,” which further indicated that there was no threshold effect.

#### Heterogeneity and inconsistency assessments

**Fig. 4:**
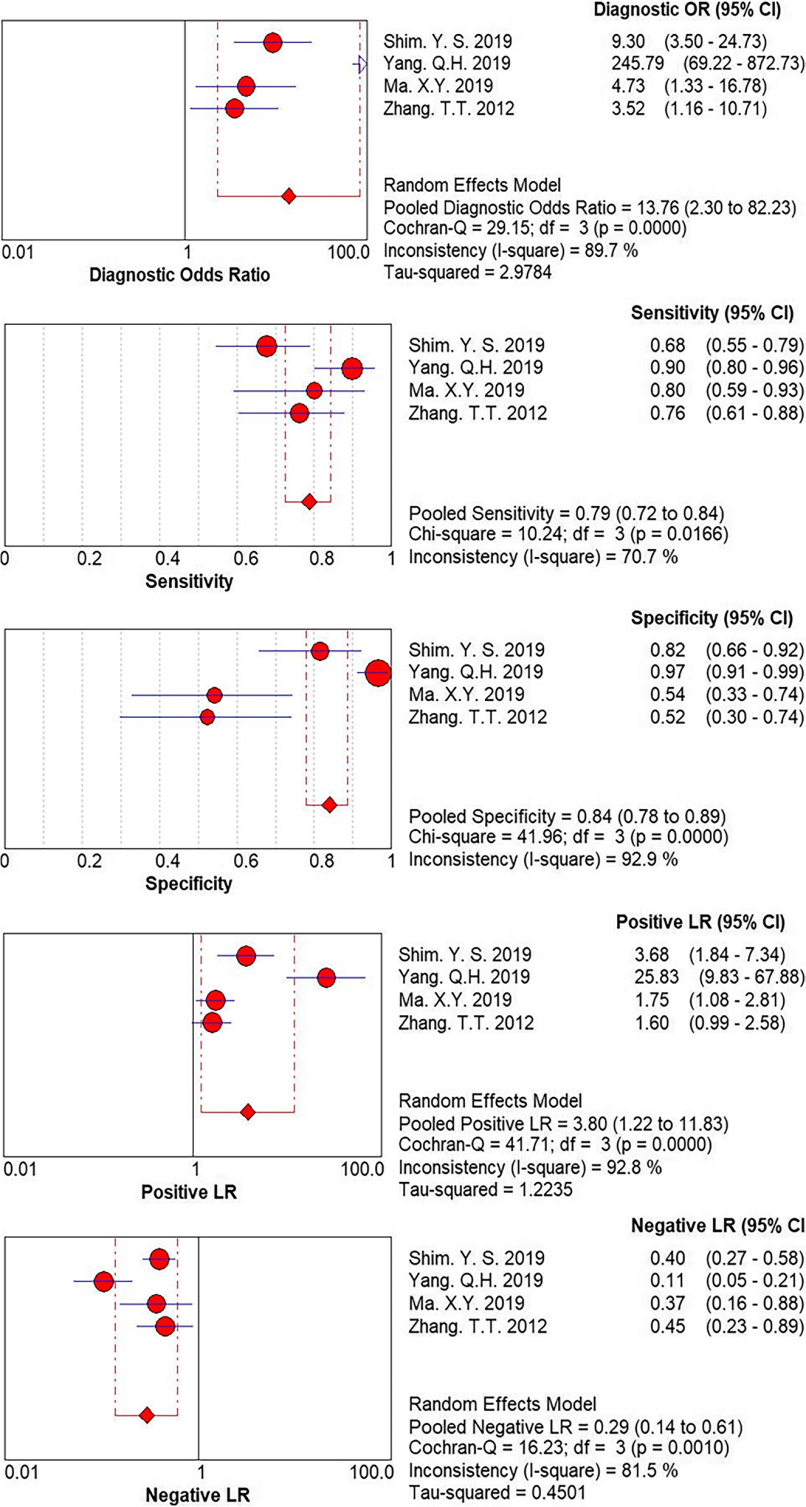
Q test and I^2^ statistic within a visual forest plot for ULH:UFSH ratio

**Fig. 5:**
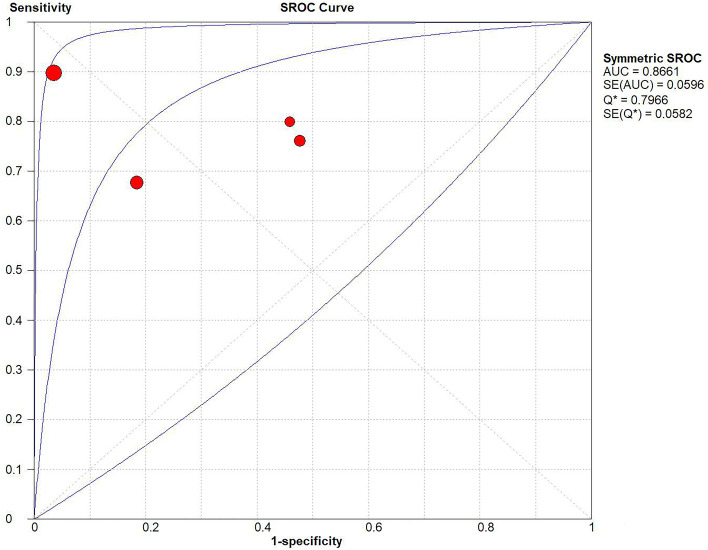
Evaluation index of diagnostic test for ULH:UFSH ratio

Heterogeneity was estimated by the Q value and I2 test. As shown in Fig. 4, *P* = 0.00 and I2 = 89.70% > 50%, indicating that heterogeneity existed. We performed a random-effects model. All forest plots of the meta-analysis are shown in Fig. 4: pooled sensitivity = 0.79 (95% CI = 0.72–0.84), pooled specificity = 0.84 (95% CI = 0.78–0.89), pooled PLR = 3.80 (95% CI = 1.22–11.83), and pooled NLR = 0.29 (95% CI = 0.14–0.61). The AUC was 0.8661 and the Q index was 0.7966 (Fig. 5).

#### Sensitivity analysis

The sensitivity analysis illustrated that two of the included studies in this meta-analysis might have caused bias, which referred to Shim’s and Yang’s studies. Moreover, we repeatedly reanalyzed the data while excluding the relevant records one by one. After deleting the data from the first record, a threshold effect was found; therefore, we did not further apply this analysis. As the data from the second record with an impact were eliminated, we obtained a *P*-value of > 0.5 and I^2^ of < 50%; therefore, we performed a fixed-effects model. The statistical results were as follows: pooled sensitivity = 0.73 (95% CI = 0.64–0.80), pooled specificity = 0.66 (95% CI = 0.55–0.76), pooled PLR = 2.19 (95% CI = 1.59–3.02), and pooled NLR = 0.40 (95% CI = 0.29–0.56). The surface under the cumulative ranking curve was 0.7820, and the Q index was 0.7203. The results were not altered when this study was removed.

#### Publication bias

Only four articles were included in the meta-analysis of the ULH:UFSH ratio; therefore, we again applied the Begg rank correlation test (*P* = 0.09) and Egger linear regression test (*P* = 0.47), which indicated that no publication bias existed.

## Discussion

Although the number of included studies was limited, we still found that some results were statistically significant. All urinary samples in the six included studies were the first-voided urine. Urinary sample collection and the GnRH stimulation test were performed on the same day. Additionally, there were few differences in the strength of the evidence among the included studies. Although the number of included articles was limited, no publication bias was found in meta-analysis of ULH and the ULH:UFSH ratio. From the six studies included in this meta-analysis, we found that compared with the GnRH stimulation test, the first-voided urinary gonadotropin test can also effectively diagnose CPP without repeated venipuncture, venous blood collection, and excessive time consumption. Furthermore, the sample collection and evaluation are more convenient, more acceptable, less expensive, and noninvasive. The test can be performed after the sample is brought to the hospital without requiring the presence of the patient. Thus, the data from this meta-analysis suggest that the first-voided urinary gonadotropin test can be used to accurately diagnose CPP.

Previous studies have indicated that first-voided urinary gonadotropins increase because of their physiological secretion during the nighttime in female patients with early puberty [17], and the present meta-analysis supports this finding. However, there are no accepted diagnostic criteria of first-voided urinary gonadotropins in pediatric endocrinology. First, all the samples were first-voided urinary samples, but the sample collection period was inconsistent across studies. The subjects in Shim et al.’s study emptied their bladder before going to bed the previous night, and first-voided urine samples were collected as soon as they woke up. There was no control for time in their study. Kobly et al.’s study used the same methods to collecte urine samples, but this study corrected all the periods by 8 h. In Yang et al.’s, Chen et al.’s and Ma et al.’s studies, there was a clear beginning time for sample collection at 20:00 h the previous night. However, the time of finishing collecting samples was not at one time, but at the time when the subjects woke up in the morning. Zhang et al.’s study used the clear time limit for sample collection, from 19:00 h to the next day at 07:00 h. Second, to avoid errors between different patients, Kolby et al. [12] used the corrected urinary gonadotropin levels along with osmolality, whereas Ma et al. [15] and Chen et al. [14] corrected the urinary gonadotropin levels along with urinary creatinine. Moreover, the test methods used across the studies in this meta-analysis were not identical. Serum gonadotropins were mainly examined by an electrochemiluminescence immunoassay, immunofluorometric assay, or chemiluminescence immunoassay. Urinary gonadotropins were measured by a dissociation-enhanced lanthanide fluorescence immunoassay, an immunofluorometric assay, a chemiluminescence immunoassay, or an immunochromatography assay. Every diagnostic approach has its own diagnostic sensitivity and specificity. Differences not only existed between the assessment of serum and urine gonadotropins but also among each of the included studies. Finally and most importantly, as mentioned above, gonadotropins in nocturnal urinary samples have been employed for CPP diagnosis for decades. In 1995, Demir et al. [18] found that urinary LH and FSH were age-related and significantly increased during puberty. However, this has not been widely adopted in clinical practice and remains an unresolved problem. In summary, the widespread use of first-voided urinary gonadotropins in the diagnosis of true precocious puberty will require a standardized examination procedure, a consistent cut-off value, and accordant urine sample collection instructions.

Although there was no fixed threshold of urinary gonadotropins in the included studies, no threshold effects existed in this meta-analysis. The meta-analysis of ULH for the diagnosis of CPP showed that the AUC was 0.8812 (sensitivity = 0.79, specificity = 0.84). In the sensitivity analysis of the outcomes in which we excluded trials with a high risk of bias one by one, we found no obvious difference in the AUC, sensitivity, or specificity. However, after excluding the data from Chen et al. [14], the diagnostic accuracy was slightly higher (AUC = 0.90, sensitivity = 0.81, specificity = 0.85), possibly because of the lower sensitivity and specificity. Regardless, ULH is a reliable indicator for the diagnosis of CPP. Similarly, the meta-analysis of the ULH:UFSH ratio for the diagnosis of CPP showed that the AUC was 0.8661 (sensitivity = 0.79, specificity = 0.84). There was no change in the sensitivity analysis of outcomes by excluding trials with a high risk of bias one by one. From this analysis, it seems apparent that the diagnostic accuracy of ULH is slightly higher than that of the ULH:UFSH ratio. No publication bias was found in this meta-analysis. In general, urinary gonadotropins can be used to effectively diagnose CPP.

First-voided urinary gonadotropin levels reflect the nighttime physiological secretion of gonadotropins in early puberty. Previous studies have shown that measurement of the nocturnal ULH and the ULH:UFSH ratio could be a proper substitute for the GnRH stimulation test [19]. In terms of the quantitative results, we found that ULH had higher diagnostic value than the ULH:UFSH ratio. However, this study had a few limitations: no large-scale study was included in the analysis; fewer than 10 studies were included, with one from Europe, one from Korea and four from China. Therefore, our results could have been skewed by the lack of studies from other parts of the world. Additionally, all the included studies were single-center studies. Thus, to better apply first-voided urinary gonadotropins in pediatric clinics, larger-scale, multicenter, and prospective studies should be conducted in the future.

## Conclusion

Although further studies are needed to establish the diagnostic value of urinary gonadotropins in CPP, our findings indicate that both the ULH level and ULH:UFSH ratio are effective and available approaches for the diagnosis of true precocious puberty from an evidence-based view.

## Supplementary Information

**Additional file 1.** Supplement 1.

**Additional file 2.** Supplement 2.

**Additional file 3.** Supplement 3.

**Additional file 4.** Supplement 4.

## Data Availability

All data analyzed during this study are included in this published article.

## Abbreviations

GnRH: Gonadotropin-releasing hormone
CPP: Central precocious puberty
ULH: Urinary luteinizing hormone
UFSH: Urinary follicle-stimulating hormone
PRISMA: Preferred Reporting Items for Systematic Reviews and Meta-Analyses
PLR: Positive likelihood ratio
NLR: Negative likelihood ratio
CI: Confidence interval
AUC: Area under the curve
