# Supplementary material for: The value of urinary gonadotropins in the diagnosis of central precocious puberty: a meta-analysis
Source: BMC Pediatr. 2022 Jul 28;22:453. doi: 10.1186/s12887-022-03481-1 (PMC9331156; doi:10.1186/s12887-022-03481-1)
Supplement: Supplementary file 1 — Additional file 1. Supplement 1. [file 12887_2022_3481_MOESM1_ESM.docx]

This is the simple introduction of the third included article, which was published in Chinese. This article’s title and abstract can be tracked from CNKI database. Some related details are as follows.

**1. Urine sample collection**

All patients emptied their bladders at 8:00 pm the day before collection of urine samples, and they neither drank nor urinated throughout the entire night. The first morning voided urine samples were collected as soon as the patients woke.

**2. Statistical analysis**

All data were processed by SPSS version 18.0 (SPSS Inc., Chicago, IL, USA). Measurement data were analyzed by the t-test and are expressed as mean ± standard deviation. Count data were analyzed by the chi-squared test and are reported as n (%). Rank count data are expressed by rank sum. A P-value of <0.05 was considered statistically significant.

**3. Results**

The detailed results are as follows.

3.1 The urinary follicle-stimulating hormone (FSH) and luteinizing hormone (LH) levels were significantly higher in girls with than without precocious puberty (P < 0.05), as shown in Table 1.

Table 1. Comparison of urinary FSH and LH levels between the two groups

| groups | cases | Urinary FSH (U/mmol) | Urinary LH (U/mmol) |
| --- | --- | --- | --- |
| precocious puberty group | n=69 | 5.78±0.59 | 2.31±0.24 |
| non-precocious puberty group | n=115 | 4.84±0.48 | 1.36±0.17 |
| *t value* |  | 6.459 | 11.385 |
| *P value* |  | 0.008 | 0.000 |

Data are presented as mean ± standard deviation.

LH, luteinizing hormone; FSH, follicle-stimulating hormone

3.2 The threshold values of the urinary FSH and LH levels in girls with and without precocious puberty were 1.60 and 5.67 U/mmol, respectively.

3.3 Comparison of the results in diagnosing female sexual development in girls with precocious puberty are shown in Table 2.

Table 2. Comparison of the results in diagnosing female sexual development in girls with precocious puberty

| Diagnostic results by means of the levels of urinary gonadotropin | | Results of 2007 version Guidelines | | Total |
| --- | --- | --- | --- | --- |
|  |  | Positive | Negative |  |
| Urinary LH | Positive | 53 | 11 | 64 |
|  | Negative | 16 | 104 | 120 |
|  | Total | 69 | 115 | 184 |
| Urinary FSH | Positive | 61 | 5 | 66 |
|  | Negative | 8 | 110 | 118 |
|  | Total | 69 | 115 | 184 |
| Urinary  LH + FSH | Positive | 62 | 4 | 66 |
|  | Negative | 7 | 111 | 118 |
|  | Total | 69 | 115 | 184 |

LH, luteinizing hormone; FSH, follicle-stimulating hormone

3.4 The sensitivity and specificity of urinary gonadotropins in the diagnosis of female precocious puberty are shown in Table 3. The calculation formulas were as follows: sensitivity = a / (a + c) and specificity = d / (b + d), where a = positive results of urinary gonadotropin and 2007 version Guidelines, b = negative result of urinary gonadotropin and positive result of 2007 version Guidelines, c = positive result of urinary gonadotropin and negative result of 2007 version Guidelines, and d = negative results of urinary gonadotropin and 2007 version Guidelines.

Table 3. Sensitivity and specificity of urinary gonadotropins in the diagnosis of female precocious puberty

|  | Sensitivity (%) | Specificity (%) | Accuracy (%) |
| --- | --- | --- | --- |
| Urinary LH | 76.81 | 90.43 | 85.33 |
| Urinary FSH | 88.41 | 95.65 | 92.93 |
| Urinary LH + FSH | 89.85 | 96.52 | 94.02 |

LH, luteinizing hormone; FSH, follicle-stimulating hormone
