# Supplementary material for: The value of urinary gonadotropins in the diagnosis of central precocious puberty: a meta-analysis
Source: BMC Pediatr. 2022 Jul 28;22:453. doi: 10.1186/s12887-022-03481-1 (PMC9331156; doi:10.1186/s12887-022-03481-1)
Supplement: Supplementary file 2 — Additional file 2. Supplement 2. [file 12887_2022_3481_MOESM2_ESM.pdf]

This is the simple introduction of the fourth included article, which was published in Chinese. This article's title and abstract can be tracked from CNKI database. Some related details are as follows.

1. Urine sample collection

Morning urine samples (MU1 and MU2) were collected for two consecutive days according to the following method. The girls emptied their bladders at 8:00 PM the day before urine sample collection, and they neither drank nor urinated during the entire night. The first morning voided urine samples were collected as soon as the girls woke. Urinary Gn (UGn and urine creatinine were measured. To eliminate the impact of urine concentration or dilution, all UGn values were corrected by urine creatinine (units of U/mmol).

2. Statistical analysis

All statistical analyses were performed using SPSS version 19.0 (IBM Corp., Armonk, NY, USA), and a P-value of <0.05 was considered statistically significant.

3. Results

The detailed results were as follows.

3.1 Comparison of UGn between MU1 and MU2

The difference in UGn between MU1 and MU2 was analyzed by a paired t-test. The P-values for urinary luteinizing hormone (ULH) and urinary follicle-stimulating hormone

(UFSH) were 0.315 and 0.924, respectively, indicating no statistical significance.

3.2 Spearman correlation analysis of serum Gn and UGn before and after Gn-releasing hormone (GnRH) stimulation test

3.2.1 Correlation between basic serum Gn and UGn in MU1 (UGn1) and UGn in MU2 (UGn2)

There were strong correlations between the basic serum LH level and the LH level in MU1 and MU2 ($r = 0.574$ and $r = 0.569$, respectively; both $P < 0.01$) and weaker correlations between the serum basal FSH level and the FSH level in MU1 and MU2 ($r = 0.419$ and $r = 0.280$, respectively; both $P < 0.01$).

3.2.2 Correlation between total 4-hour UGn after GnRH stimulation test and UGn1 and UGn2

The correlations between the 4-hour ULH level and the ULH level in MU1 (ULH1) and MU2 (ULH2) were 0.609 and 0.657, respectively (both $P < 0.01$). The correlations between the 4-hour UFSH level and the UFSH level in MU1 (UFSH1) and MU2 (UFSH2) were 0.445 and 0.412, respectively (both $P < 0.01$). Thus, all correlations were strong.

3.2.3 Correlation between total 4-hour UGn and serum Gn peak after GnRH stimulation test

The correlations of LH and FSH between the 4-hour and peak concentrations were 0.719 and 0.403, respectively (both $P < 0.01$), indicating a strong correlation for both.

3.2.4 Correlation between serum LH:FSH ratio and 4-hour ULH:UFSH ratio after GnRH stimulation test

The correlation between these values was 0.591 ($P < 0.01$), exhibiting a strong correlation.

3.3 Comparison of hormone levels among Tanner stages II, III, and IV

The serum basal Gn, UGn1, UGn2, serum Gn peak, and LH:FSH ratio after the GnRH stimulation test as well as the UGn and 4-hour ULH:UFSH ratio were compared among different stages of breast development (Table 1) by one-way analysis of variance.

3.3.1 Serum basal Gn, LH peak, and LH:FSH ratio

The differences among the three stages were statistically significant ($P < 0.05$). In particular, the P-value of ULH1, ULH2, total 4-hour ULH, and the ULH:UFSH ratio after the GnRH stimulation test were <0.01.

3.3.2 Serum basal LH, LH peak, LH:FSH ratio, and LH in MU1

The differences between Tanner stage II and the other two stages were statistically significant ($P < 0.05$). The differences in ULH2, 4-hour ULH, and the ULH:UFSH ratio between Tanner stages II and III were also statistically significant ($P < 0.05$).

3.4 Evaluation of serum Gn and UGn among Tanner stages according to receiver operating characteristics (ROC) curve analysis

3.4.1 Clinical value of basic serum LH and morning ULH

The areas under the ROC of basic serum LH, basic serum FSH, ULH1, ULH2, FSH in MU1 (UFSH1), and FSH in MU2 (UFSH2) were 0.692, 0.609, 0.655, 0.714, 0.634, and 0.492, respectively (Figure 1). The serum LH cut-off point was 0.155 with a sensitivity of 80.8% and specificity of 54.2%. The ULH1 cut-off point was 2.355 with a sensitivity of 73.1% and specificity of 56.6%. The ULH2 cut-off point was 1.43 with a sensitivity of 88.5% and specificity of 45.8%.

3.4.2 Clinical value of serum peak LH, LH:FSH ratio, LH, and ULH:UFSH ratio in total

4-hour urinary sample

The areas under the ROC of the serum peak LH, peak FSH, LH:FSH ratio, total 4-hour ULH, UFSH, and ULH:UFSH ratio were 0.728, 0.427, 0.748, 0.741, 0.399, and 0.741, respectively (Figure 2). The clinical value was higher for the serum peak LH, serum LH:FSH ratio, and ULH:UFSH ratio in the total 4-hour urinary sample. The serum peak LH cut-off point was 6.2 with a sensitivity of 84.6% and specificity of 59.0%. The serum LH:FSH ratio cut-off point was 0.845 with a sensitivity of 80.8% and specificity of 61.4%. The total 4-hour ULH cut-off point was 5.97 with a sensitivity of 69.2% and specificity of 80.7%. The total 4-hour ULH:UFSH ratio cut-off point was 0.79 with a sensitivity of 65.4% and specificity of 80.7%.

3.5 Analysis of ROC curve

The diagnostic criteria for central precious puberty indicate that the serum LH:FSH ratio should be >0.6 after the GnRH stimulation test. The value of UGn1, UGn2, UGn, and the ULH:UFSH ratio in total 4-hour urine in evaluation of a serum LH:FSH ratio of >0.6 was as follows. ULH1, ULH2, 4-hour urinary ULH, and the ULH:UFSH ratio had greater evaluation value, and the areas under the ROC were 0.781, 0.776, 0.779, and 0.812, respectively (Figure 3), indicating that these indices can help to distinguish premature thelarche and central precious puberty. The ULH1 cut-off point was 1.58 with a sensitivity of 79.4% and specificity of 71.7%. The ULH2 cut-off point was 2.11 with a sensitivity of 71.4% and specificity of 76.1%. The total 4-hour ULH cut-off point was 3.27 with a sensitivity of 82.5% and specificity of 71.7%. The total 4-hour ULH:UFSH ratio cut-off point was 0.49 with a sensitivity of 69.8% and specificity of 84.8%.

Table 1. Comparison of serum Gn and UGn among different Tanner stages

Tanner stage	Serum(U/L)				Serum	Morning urine (U/mmol)				4-hour urine (U/mmol)		4-hour urine ULH: UFSH ratio
	Basal LH	Basal FSH	Peak LH	Peak FSH	LH:FSH ratio	ULH1	ULH2	UFSH1	UFSH2	ULH	UFSH	
II	0.43± 0.60[1)]	2.52± 1.49	7.43± 7.07[1)]	10.07 ±4.61	0.81± 0.65[1)]	2.28± 2.26[1)]	2.10± 1.69[2)]	4.81± 3.77	5.49± 5.55	3.89± 2.65[2)]	9.46± 5.56	0.60± 0.87
III	1.22± 1.40	3.10± 1.66	16.03± 12.41	9.29± 5.10	1.74± 1.07	4.39± 3.66	4.18± 3.01	5.89± 5.31	4.45± 2.49	8.37± 7.92	7.48± 4.26	1.28± 1.13
IV	2.20± 1.83	4.30± 2.83	17.96± 10.72	7.80± 3.40	2.18± 1.36	5.44± 2.93	3.81± 1.32	8.39± 7.28	5.76± 2.90	4.83± 1.45	10.05 ±7.23	0.69± 0.45
F	13.17	3.37	11.25	0.58	17.10	7.93	10.53	1.75	0.45	9.81	1.42	5.31
P	<0.01	0.038	<0.01	0.562	<0.01	0.001	<0.01	0.179	0.638	<0.01	0.245	0.006

[1)]Compared with the other two stages, P < 0.05

[2)] Compared with Tanner stage III, P < 0.05

LH, luteinizing hormone; FSH, follicle-stimulating hormone; ULH1, LH in morning urine 1; ULH2, LH in morning urine 2; UFSH1, FSH in morning urine 1; UFSH2, FSH in morning urine 2; ULH, LH in 4-hour urine; UFSH, FSH in 4-hour urine.

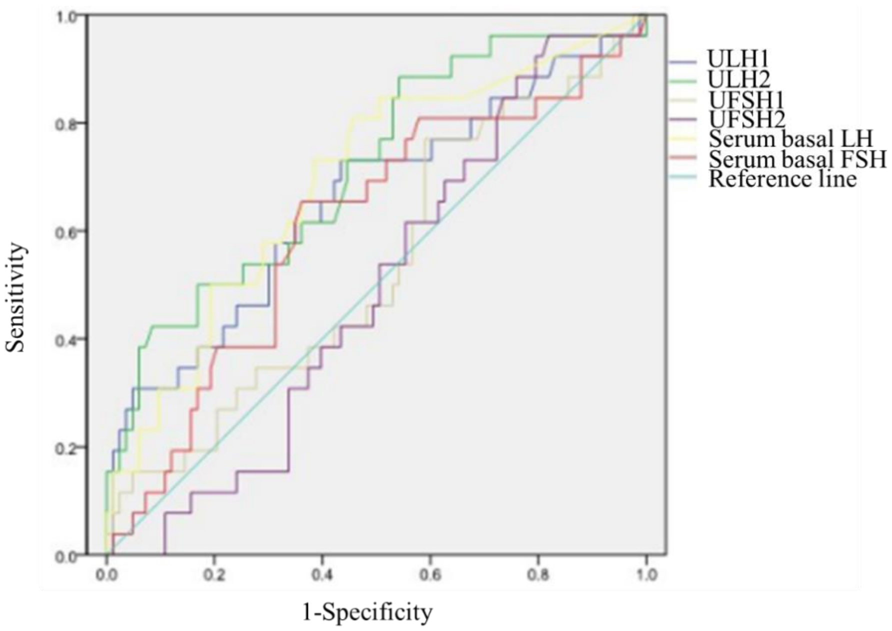

Figure 1. Receiver operating characteristics curve analysis of the clinical value of serum and urinary gonadotropins in distinguishing Tanner stages II and III. LH, luteinizing hormone; FSH, follicle-stimulating hormone; ULH1, LH in morning urine 1; ULH2, LH in morning urine 2; UFSH1, FSH in morning urine 1; UFSH2, FSH in morning urine 2.

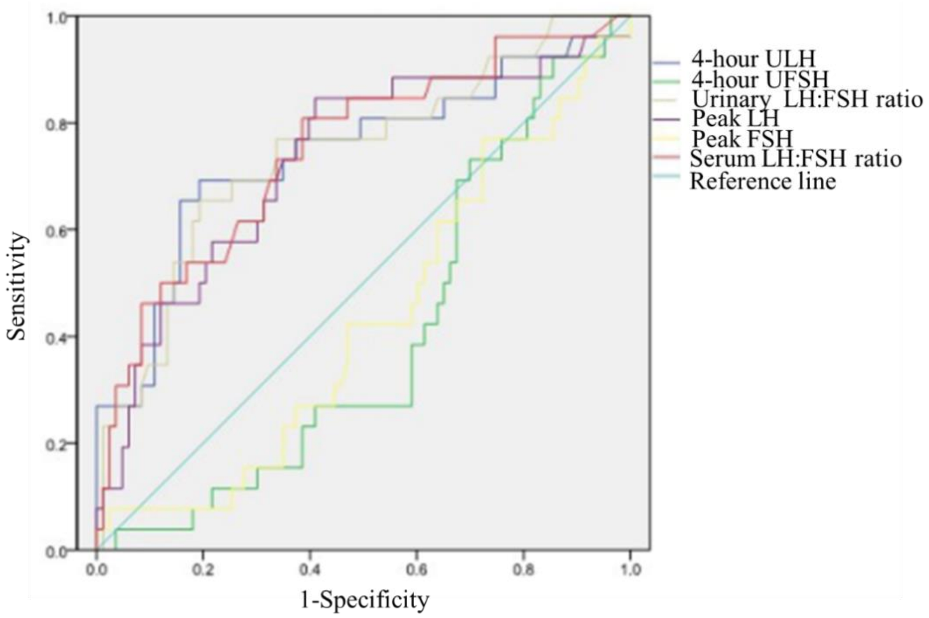

Figure 2. Receiver operating characteristics curve analysis of the clinical value of serum peak gonadotropins and 4-hour urinary gonadotropins after gonadotropin-releasing hormone stimulation test in distinguishing Tanner stages II and III. LH, luteinizing hormone; FSH, follicle-stimulating hormone; ULH, LH in 4-hour urine; UFSH, FSH in 4-hour urine.
