# Supplementary material for: The value of urinary gonadotropins in the diagnosis of central precocious puberty: a meta-analysis
Source: BMC Pediatr. 2022 Jul 28;22:453. doi: 10.1186/s12887-022-03481-1 (PMC9331156; doi:10.1186/s12887-022-03481-1)
Supplement: Supplementary file 3 — Additional file 3. Supplement 3. [file 12887_2022_3481_MOESM3_ESM.pdf]

This is the simple introduction of the fifth included article, which was published in Chinese. This article's title and abstract can be tracked from CNKI database. Some related details are as follows.

1. Girls with premature thelarche

Premature thelarche (PT) is defined as the development of breasts before the age of 8 years in girls. Such patients do not show accelerated growth, and their luteinizing hormone-releasing hormone (LHRH) stimulation test results do not reach the criteria for central precocious puberty (CPP). Classic PT is diagnosed when there is no non-regression or continuous enlargement of mammary gland nuclei, no accelerated growth or continued progression of other sexual characteristics, and LHRH stimulation test results that do not meet the criteria for CPP throughout a 6-month follow-up. In contrast, transitional PT is diagnosed when the breast progressively enlarges again or enlarges after shrinking and no longer shrinks again after enlarging many times, accelerated growth and maturation of other sexual signs occurs, and LHRH stimulation test results do not reach the diagnostic criteria for CPP throughout a 6-month follow-up.

2. Urine analyses

The girls emptied their bladders at 8:00 PM the day before the urine samples were collected, and they neither drank nor urinated throughout the entire night. The first morning voided urine samples were then collected. The urinary LH and

follicle-stimulating hormone (FSH) levels were examined with an immunochromatographic interpretation recorder. The urinary creatinine level was measured in urine samples to adjust for urinary LH and FSH, and the LH:FSH ratio was calculated.

3. Statistical analyses

All statistical calculations were performed using SPSS version 13.0 (SPSS Inc., Chicago, IL, USA). Variables are reported as mean ± standard deviation. The diagnostic validity of each variable was evaluated by receiver operating characteristic (ROC) curve analysis. A P-value of <0.05 was considered statistically significant.

4. Results

The detailed results are as follows.

4.1 Diagnostic results

In total, 112 girls with breast development before 8 years of age underwent both an LHRH stimulation test and urinary LH and urinary FSH assays. Of these 112 girls, 53 were diagnosed with CPP (their LHRH stimulation test showed a peak LH level of ≥5 IU/L) and started treatment immediately. Ten girls were diagnosed with peripheral precocious puberty (5 had McCune-Albright syndrome and 5 had congenital adrenal hyperplasia) and were thus excluded from the study. The remaining 49 girls were diagnosed with PT and were observed for 6 months with no therapy. They were then divided into two groups (classic PT group and transitional PT group) for comparison

of clinical signs and laboratory test results. There was no significant difference in either the serum basal or peak LH and FSH levels between the two groups (Table 1).

4.2 Correlations of urinary LH and FSH with serum LH and FSH

Spearman correlation analyses were conducted for urinary LH and FSH and serum LH and FSH. The results showed that serum LH had a positive correlation with morning urinary LH ($r = 0.24$, $P < 0.05$) (Figure 1).

4.3 Children with PT

In total, 49 girls with PT underwent another LHRH stimulation test after 6 months of follow-up without therapy. Among them, 25 girls were diagnosed with transitional PT because there was enlargement of the ovaries, no shrinkage of the mammary gland, no bone age maturation, and serum LH level were ≥5 IU/L. The remaining girls were diagnosed with classic PT (Table 2).

There were no significant differences in serum basal and peak LH and FSH or in the LH:FSH ratio between girls with classic PT and those with transitional PT at their first time for LHRH stimulation test (Table 1).

4.4 Urinary LH and FSH levels and LH:FSH ratio in girls with PT

The examination of morning urinary samples showed that the LH level in girls with classic and transitional PT was 1.30 and 2.35 IU/L, respectively, without a significant difference. However, the FSH level was slightly lower in girls with transitional PT than in those with classic PT, again with no significant difference. The morning urinary LH/FSH ratio in girls with classic PT and transitional PT at their first visit was 0.50 ± 0.35 and 1.16 ± 1.08, respectively, and the difference was statistically

significant (P = 0.02,Table 1).

4.5 Diagnostic value of urinary LH:FSH ratio, serum peak LH level, and serum peak LH:FSH ratio in distinguishing classic and transitional PT

Comparison of the urinary LH:FSH ratio, serum peak LH level, and serum peak LH:FSH ratio in differentiating classic PT from transitional PT was performed by ROC curve analysis, and the best diagnostic cut-off value was determined at the same time. The ROC curve analysis showed that the LH:FSH ratio was the best diagnostic index, with a cut-off value of 0.512, sensitivity of 80%, and specificity of 56%. The areas under the ROC curve of the serum peak LH level and serum peak LH:FSH ratio were both <0.7. The areas under the ROC curve of the three indexes were significantly different (P < 0.05) (Table 3).

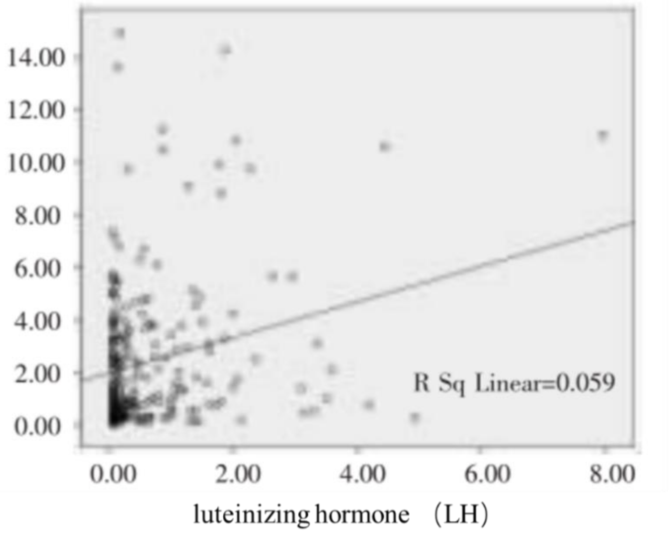

Figure 1. Correlation between serum LH and morning urinary LH

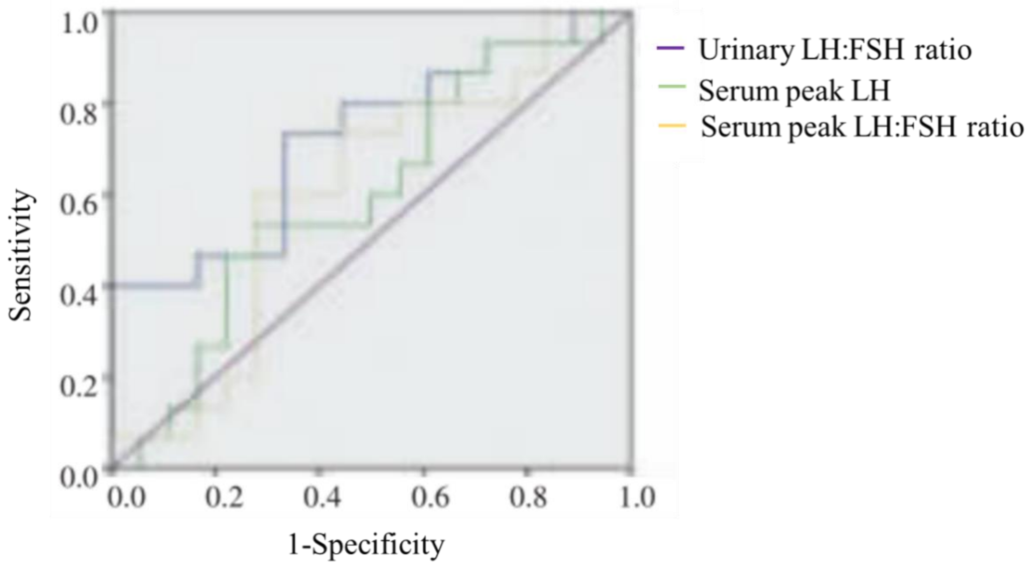

Figure 2. ROC curve for analysis of urinary LH:FSH ratio, serum peak LH level, and serum peak LH:FSH ratio. LH, luteinizing hormone; FSH, follicle-stimulating hormone

Table 1. Serum and urinary LH, FSH, and LH:FSH ratio in girls with classic PT and transitional PT

	classical PT	transitional PT	P value
Serum basal LH (IU/L)	0.14±0.05	0.17±0.04	0.57
Serum peak LH (IU/L)	2.67±1.59	3.14±1.29	0.35
Serum basal FSH (IU/L)	1.48±0.67	2.02±1.23	0.06
Serum peak FSH (IU/L)	10.45±6.93	10.50±5.04	0.97
Serum peak LH:FSH ratio	0.38±0.12	0.39±0.11	0.93
Morning urinary LH (IU/L)	1.30±0.27	2.35±0.99	0.12
Morning urinary FSH (IU/L)	3.45±1.20	2.78±1.52	0.49
Morning urinary LH:FSH ratio	0.50±0.35	1.16±1.08	0.02
Serum peak LH after 6-month (IU/L)	2.56±1.09	5.75±0.49	<0.01
Serum peak FSH after 6-month (IU/L)	10.43±5.50	8.26±0.88	0.10

Data are presented as mean ± standard deviation.

PT, premature thelarche; LH, luteinizing hormone; FSH, follicle-stimulating hormone

Table 2. Conditions of 49 girls with PT

	classical PT (n=24)	transitional PT (n=25)
Breast budding age (years)	5.68±1.60	6.14±1.51
Age of first visit (years)	6.11±1.11	6.76±1.02
Bone age (years)	6.01±1.80	7.91±1.52
Height-SDS	0.66±0.33	1.02±0.71

BMI (kg/m^2)	12.80±2.64	16.00±2.22

Data are presented as mean ±standard deviation.
PT, premature thelarche; BMI, body mass index

Table 3. Diagnostic value of urinary LH:FSH ratio, serum peak LH level, and serum peak LH:FSH ratio in differentiating classic PT from transitional PT and their best cut-off value

	Morning urinary LH:FSH ratio	Serum peak LH:FSH ratio	Serum peak LH
The best cutoff value	≥0.512	≥0.290	≥0.596
The area under ROC curve	0.722	0.640	0.596
Sensitivity	80.0%	60.0%	53.3%
Specificity	55.6%	73.2%	73.2%

LH, luteinizing hormone; FSH, follicle-stimulating hormone; PT, premature thelarche; ROC, receiver operator characteristics
