# Supplementary material for: The value of urinary gonadotropins in the diagnosis of central precocious puberty: a meta-analysis
Source: BMC Pediatr. 2022 Jul 28;22:453. doi: 10.1186/s12887-022-03481-1 (PMC9331156; doi:10.1186/s12887-022-03481-1)
Supplement: Supplementary file 4 — Additional file 4. Supplement 4. [file 12887_2022_3481_MOESM4_ESM.pdf]

This is the simple introduction of the sixth included article, which was published in Chinese. This article's title and abstract can be tracked from CNKI database. Some related details are as follows.

1. Serum and urinary gonadotropin (Gn) levels and detectability

In girls with central precocious puberty (CPP), the serum peak luteinizing hormone (PLH) and serum peak follicle-stimulating hormone (PFSH) were 22.89 ± 17.31 and 15.43 ± 7.12 IU/L, respectively, and the nocturnal spontaneous urinary LH and urinary FSH were 0.303 ± 0.345 and 3.233 ± 2.250 IU, respectively. In girls with non-CPP, the PLH and serum PFSH were 2.78 ± 1.21 and 11.11 ± 5.12 IU/L, respectively, and the nocturnal spontaneous urinary LH and urinary FSH were 0.123 ± 0.294 and 1.941 ± 2.956 IU/L, respectively. The levels and detectability of spontaneous serum Gn and urinary Gn are shown in Table 1.

2. Correlation of nocturnal spontaneous urinary Gn, spontaneous serum Gn, and serum peak Gn and their receiver operating characteristic (ROC) curve analysis in girls with CPP

All of the above results are summarized in Table 2. When the PLH was ≥4.86 IU/L, the sensitivity and specificity for the diagnosis of CPP were 92.9% and 100%, respectively, and the area under the ROC curve was 0.956. When the PLH:PFSH ratio was ≥0.479, the sensitivity and specificity for the diagnosis of CPP were 88.1% and 95.2%, respectively, and the area under ROC curve was 0.926.

Table 1. Levels and detectability of spontaneous serum Gn and urinary Gn

		Nocturnal spontaneous serum		Diurnal spontaneous serum		Nocturnal spontaneous urinary	
		LH	FSH	LH	FSH	LH	FSH
CPP (n=42)	Level (IU/L)	3.29±4.07	4.26±2.63	1.73±2.86	4.27±4.00	0.80±1.38	7.93±8.53
	Dectability (%)	85.7	100.0	81.0	100.0	69.0	100.0
Non-CPP (n=21)	Level (IU/L)	1.06±1.45	3.03±2.24	0.60±0.86	2.31±1.45	0.33±0.76	6.09±8.21
	Dectability (%)	85.7	90.5	61.9	90.5	33.3	95.2

Levels are presented as mean ± standard deviation.

Detectability: the percentage of samples with a level of >0.2 IU/L.

Gn, gonadotropin; CPP, central precocious puberty; LH, luteinizing hormone; FSH, follicle-stimulating hormone

Table 2. Correlation between serum and urinary gonadotropin and receiver operator characteristic curve analysis in analyzing the start of hypothalamic-pituitary-gonadal axis activity for girls with central precocious puberty

	Nocturnal spontaneous serum		Diurnal spontaneous serum		Nocturnal spontaneous urinary	
	SLH (IU/L)	SLH/SFSH (IU/L)	SLH (IU/L)	SLH/SFSH (IU/L)	LH (IU)	LH/FSH (IU)
Correlation coefficient a	0.423**	0.441*	0.514*	0.479*	0.597*	0.464*
Correlation coefficient b	0.585*	0.495*	0.546*	0.361**	-	-
Cutoff value	1.39	0.38	0.97	0.059	0.113	0.044
Sensitivity (%)	54.8	52.4	42.9	92.9	71.4	76.2
Specificity (%)	85.7	76.2	90.5	23.8	90.5	52.4
Area under ROC curve	0.654	0.592	0.680	0.556	0.825	0.595

Correlation coefficient a: correlation coefficient with serum peak gonadotropin in GnRH stimulation test

Correlation coefficient b: correlation coefficient with nocturnal spontaneous urinary gonadotropin

LH, luteinizing hormone; FSH, follicle-stimulating hormone; SLH, serum LH; SFSH, serum FSH; ROC, receiver operator characteristic

*P < 0.001; **P < 0.01
